# Prevalence of abnormal glucose metabolism in pediatric acute, acute recurrent and chronic pancreatitis

**DOI:** 10.1371/journal.pone.0204979

**Published:** 2018-10-31

**Authors:** Maisam Abu-El-Haija, Lindsey Hornung, Lee A. Denson, Ammar Husami, Tom K. Lin, Kristal Matlock, Jaimie D. Nathan, Joseph J. Palermo, Tyler Thompson, C. Alexander Valencia, Xinjian Wang, Jessica Woo, Keijan Zhang, Deborah Elder

**Affiliations:** 1 Division of Pediatric Gastroenterology, Hepatology and Nutrition, Cincinnati Children’s Hospital Medical Center, Cincinnati, Ohio, United States of America; 2 Department of Pediatrics, University of Cincinnati College of Medicine, Cincinnati, Ohio, United States of America; 3 Division of Biostatistics and Epidemiology, Cincinnati Children’s Hospital Medical Center Cincinnati, Ohio, United States of America; 4 Division of Human Genetics, Cincinnati Children’s Hospital Medical Center, Cincinnati, Ohio, United States of America; 5 Division of Pediatric Endocrinology, Cincinnati Children’s Hospital Medical Center, Cincinnati, Ohio, United States of America; 6 Division of Pediatric General and Thoracic Surgery, Cincinnati Children’s Hospital Medical Center, Cincinnati, Ohio, United States of America; University of Szeged, HUNGARY

## Abstract

Type 3C Diabetes, or diseases of the exocrine pancreas has been reported to occur in approximately 30% of adult patient with pancreatitis. The incidence of glucose abnormalities or risk factors that may predict the development of abnormal glucose in the pediatric pancreatitis population is not known. We performed a retrospective chart review from 1998–2016 for patients who carry the diagnosis of acute pancreatitis (AP), acute recurrent pancreatitis (ARP), and chronic pancreatitis (CP). We extracted glucose values, HbA1c%, and data from oral glucose tolerance and mixed meal testing with timing in relation to pancreatic exacerbations. Patient characteristic data such as age, gender, body proportions, family history of pancreatitis, exocrine function and genetic mutations were also assessed. Abnormal glucose was based on definitions put forth by the American Diabetes Society for pre-diabetes and diabetes. Fifty-two patients had AP and met criteria. Of those, 15 (29%) had glucose testing on or after the first attack, 21 (40%) were tested on or after the second attack (in ARP patients) and 16 (31%) were tested after a diagnosis of CP. Of the patients tested for glucose abnormalities, 25% (13/52) had abnormal glucose testing (testing indicating pre-DM or DM as defined by ADA guidelines. A significantly higher proportion of the abnormal glucose testing was seen in patients (85%, 11/13) with a BMI at or greater than the 85^th^ percentile compared to the normal glucose patients (28%, 11/39) (p = 0.0007). A significantly higher proportion of the abnormal glucose patients (77%, 10/13) had SAP during the prior AP episode to testing compared to the 10% (4/39) of the normal glucose patients (p<0.0001). Older age at DM testing was associated with a higher prevalence of abnormal glucose testing (p = 0.04). In our patient population, a higher proportion of glucose abnormalities were after the second episode of pancreatitis, however 62% (8/13) with abnormalities was their first time tested. We identified obesity and having severe acute pancreatitis (SAP) during the prior AP episode to testing could be associated with abnormal glucose. We propose that systematic screening for abnormal glucose after the first episode of acute pancreatitis in order to better establish the timing of diabetes progression.

## Introduction

Acute pancreatitis (AP) in children is increasingly recognized, and growing in incidence[1]. A subset of children progress to acute recurrent pancreatitis (ARP) and chronic pancreatitis (CP), disease entities which have recently been defined by the International Study Group of Pediatric Pancreatitis: In Search for a Cure (INSPPIRE)[2]. Adults with AP are at an increased risk of developing prediabetes (pre-DM) and diabetes mellitus (DM), particularly Type 3c DM, after pancreatitis but little is known about glucose intolerance after AP in children[3]. Children are not “mini-adults”, given the different etiologies of AP. Biliary and alcohol are the predominant etiologies in adult AP[4, 5], while pediatric AP is more often secondary to biliary, metabolic, genetic or anatomic abnormalities[6–8], making it difficult to extrapolate risk models and therapies for DM related to AP from adults to pediatrics[9]. Due to a paucity of pediatric studies, significant knowledge gaps remain regarding risks and complications of AP, ARP and CP such as diabetes. Understanding the natural history will lead to the generation of risk models to help predict complications, and eventually the development of therapies that may impede disease progression.

Adult studies show that up to 40% of patients develop abnormal glucose metabolism after a single episode of AP[10], with a 2.5 fold increased DM risk compared to the general population[11]. DM is a disease with negative implications on the child’s health[12]. DM was shown to occur post CP in children[13]; however, there are no studies that examine risk of DM post AP or ARP. Adult AP studies have focused on developing risk scores for DM[14], but these efforts are lacking in pediatrics. Moreover, there are no pediatric guidelines for pre-DM or DM risk assessment post AP. It is important to characterize different types of DM, as the pancreatogenic or Type 3C DM (T3cDM) which has different implications and therapies from Type 1, 2 or cystic fibrosis-related diabetes (CFRD)[3, 15]. It is unclear if post AP pre-DM is within the continuum of T3cDM, Type 2 or Type 1 or even a combination. Currently, DM is under-recognized, and providers lack the tools to identify children at risk after AP. Additional work is needed to alert us to children in need for specialized evaluation and therapy for DM.

T3cDM has been linked to increased risk of pancreatic adenocarcinoma emphasizing the need for early recognition, starting by alerting physicians of at-risk patients [3, 15, 16]. Understanding risk factors for pre-DM and DM in children post pancreatitis, will shed light on pathophysiology for future directed therapies and precision medicine [17–19].

The goal of our study is to identify key clinical and genetic factors that are associated with endocrine insufficiency (pre-DM and DM) following pancreatitis in children.

## Methods

This study was approved by the institutional review board (IRB # 2016–9073). We performed a retrospective chart review from 1998–2016 for patients diagnosed with AP, ARP or CP and had glucose-related testing. Consent was waived under this IRB protocol. We extracted glucose values, hemoglobin A1c (HbA1c) and data from oral glucose tolerance and mixed meal testing. Patient clinical characteristics such as age, gender, body proportions, family history of pancreatitis, exocrine function testing, characteristics of the first AP attack and subsequent ARP and CP episodes as well as genetic variants were assessed. Abnormal glucose was based on definitions by the American Diabetes Association (ADA) for pre-diabetes and diabetes (pre-DM criteria: fasting blood glucose [FBG] ≥100 mg/dL, HbA1c ≥5.7%, or 2-hour glucose testing of ≥140 mg/dL; DM criteria: FBG ≥126 mg/dL, HbA1c ≥6.5%, or 2-hour or random glucose testing of ≥200 mg/dL)**[20]**. Severe acute pancreatitis (SAP) was defined as per the established new classification and definition of AP[21].

## Genetic testing

Genetic testing was performed by clinical laboratories; whereby, pancreas-related genes (*PRSS1*, *CTRC*, *CFTR* and *SPINK1*) were analyzed by Sanger sequencing or next-generation sequencing. Briefly, genomic DNA was isolated from the patient’s specimen using a standard kit and quantified. For Sanger sequencing, regions of the genomic DNA for which testing was requested were selectively amplified through the PCR followed by Sanger sequencing on a genetic analyzer (ABI). For genes analyzed by next-generation sequencing, analysis was done by doing variant alignment (BAM), variant calling (VCF) and variant annotation, and report generation by proprietary methods.

## Variant classification

We employed the American College of Medical Genetics guidelines for the classification of Mendelian (*PRSS1*), susceptibility (*CTRC*) and Mendelian/susceptibility (*CFTR*, *SPINK1*) gene variants as the most common genes involved in ARP and CP [22, 23] For Mendelian genes, variants were evaluated using evidence from population data (rare variants in the Genome Aggregation Database established by Broad Institute (http://gnomad.broadinstitute.org/)) and gene/disease-specific databases (http://www.pancreasgenetics.org/), computational prediction tools (SIFT, POLYPHEN-2 and Grantham scores), our in-house variant database, presence in multiple publicly (ClinVar) and commercially available (Human Gene Mutation Database, HGMD, Online Mendelian Inheritance in Man, OMIM) mutation databases and appropriate scientific literature (PUBMED) (functional data) and parental testing (segregation data). More specifically, special attention was focused on rare variants (<1%) with potentially damaging effects assessed by variant type predicted to cause disease (frameshift, start loss and nonsense changes, splice site mutations) and missense changes with pathogenic scores as predicted by SIFT, POLYPHEN-2 and Grantham scores. Moreover, variants reported in the Human Gene Mutation Databases were also assessed by reviewing the primary literature. Based on the evidence available for each variant, the utilization of specific standard terminology—“pathogenic”, “likely pathogenic”, “uncertain significance or variants of unknown clinical significance” (VUCS), “likely benign” and “benign”—to describe variants identified in genes that cause Mendelian disorders was employed. VUCS remained its own category and *likely benign* and *benign* variants were not included in the analysis.

For non-Mendelian genes, variants were queried against ClinVar, HGMD, OMIM and the pancreas genetics databases to establish whether they had previously reported. If a specific variant had been previously reported in genome-wide association or case-control studies, statistical significance was noted to be included as an “established risk factor” (GWAS *p* value <5X10^-8^; case-control with odds Ratio (OR) >1 and *p* value <0.05 with confidence Interval >1). Conversely, if the statistical thresholds were not meet, variants were classified as benign and not included. However, variants that had not been previously reported or variants with unavailable *p* values were classified as *uncertain risk factors*. Unknown significance, likely pathogenic, pathogenic, established risk and uncertain risk variants were confirmed by Sanger sequencing.

## Statistical analysis

Data were analyzed using SAS, version 9.4 (SAS Institute, Cary, NC). Due to sample sizes and the distribution of variables, continuous data were summarized as medians with 25^th^ and 75^th^ percentiles (Interquartile range, IQR) while categorical data were summarized as frequency counts with percentages. Fisher’s exact tests were used for group comparisons of categorical variables. For continuous data, non-parametric Wilcoxon-Mann-Whitney tests were used to compare characteristics between groups. *P* <0.05 was considered statistically significant for analyses.

## Results

We identified 90 patients with known pancreatitis dates and diabetes testing during the study period. Patients who had glucose data after a diagnosis of cystic fibrosis (n = 28) or underwent a partial pancreatic resection or total pancreatectomy (n = 10) were excluded from the analysis. A total of 52 patients with available glucose testing data over the study period were included in the analysis. Demographic data is presented in Table 1.

**Table 1:** Demographic and clinical data of patients with AP, ARP, CP and available glucose testing for glucose and HbA1c.

	All N = 52
**Age 1**^**st**^ **AP attack (years)**	10.3 (6.2, 14.0)
**Age at DM testing (years)**	12.8 (9.2, 16.1)
**Time 1**^**st**^ **AP attack to DM testing (years)**	1.4 (0.6, 2.6)
**Gender (male)**	26 (50%)
**Family history of pancreatitis (yes)**	15 (29%)
**Exocrine pancreatic insufficiency**	11/30 (37%)
**BMI ≥ 85**^**th**^ **percentile**	22 (42%)
**SAP prior episode to testing (yes)**	14 (27%)
**Genetic testing done**	45 (87%)
**Patient with genetic testing positive**	31/45 (69%)
**ARP at DM testing (yes)**	30 (58%)
**Time ARP to DM testing (years)**	0.8 (0.2, 1.6)
**CP at DM testing (yes)**	19 (37%)
**Time CP to DM testing (years)**	0.2 (0.0, 0.9)

BMI (body mass Index), SAP (Severe Acute Pancreatitis), ARP (Acute Recurrent Pancreatitis), DM (Diabetes Mellitus), CP (Chronic Pancreatitis). Data presented as median (25^th^, 75^th^ percentile) and frequency (%).

### Characteristics of the AP attacks

From the 52 patients, 15 (29%) had glucose testing on or after the first attack, 21 (40%) were tested on or after the second attack (in ARP patients) and 16 (31%) were tested after a diagnosis of CP. Of the patients tested for glucose abnormalities, 25% (13/52) had abnormal glucose testing (testing indicating pre-DM or DM as defined by ADA guidelines).[20]

Abnormal glucose testing was found in 15% (2/13) of patients after their first known attack of AP, 46% (6/13) on or after the second AP episode (3 on or after second AP, 1 after third AP, 2 after fourth AP attack) and prior to meeting criteria for CP, and 38% (5/13) after diagnosis of CP. However, for 62% (8/13) with abnormalities, it was their first glucose testing on record, and for 7 of those 8 patients it was their only glucose test on record, 5 had normal glucose testing prior to the abnormal value.

A significantly higher proportion of the abnormal glucose testing was seen in patients (85%, 11/13) with a BMI at or greater than the 85^th^ percentile compared to the normal glucose patients (28%, 11/39) (p = 0.0007). A significantly higher proportion of the abnormal glucose patients (77%, 10/13) had SAP during the prior AP episode compared to the 10% (4/39) of the normal glucose patients (p<0.0001). Older age at DM testing was associated with a higher prevalence of abnormal glucose testing (p = 0.04). The presence of exocrine pancreatic insufficiency prior to glucose testing or positive genetic testing did not differ between groups. Group comparisons are shown in Table 2.

**Table 2:** Group comparison of normal glucose testing and abnormal glucose testing patients.

	Normal Testing N = 39	*Abnormal Testing N = 13	P-value
**Age 1**^**st**^ **AP attack (years)**	8.9 (5.2, 13.3)	13.5 (11.3, 14.3)	0.16
**Age at DM testing (years)**	11.9 (8.9, 16.4)	15.0 (13.8, 15.8)	**0.04**
**Time 1**^**st**^ **AP attack to DM testing (years)**	1.2 (0.5, 2.3)	1.5 (0.7, 4.5)	0.56
**Gender (male)**	17 (44%)	9 (69%)	0.20
**Family history of pancreatitis (yes)**	27 (69%)	10 (77%)	0.73
**Exocrine pancreatic insufficiency**	10/25 (40%)	1/5 (20%)	0.63
**BMI ≥ 85**^**th**^ **percentile**	11 (28%)	11 (85%)	**0.0007**
**SAP during prior AP episode (yes)**	4 (10%)	10 (77%)	**<0.0001**
**Days from SAP prior episode to glucose testing**	224.5 (97.0, 572.5) *n = 4*	242.0 (68.0, 466.0) *n = 10*	0.84
**Genetic testing done**	33 (85%)	12 (92%)	0.66
**Genetic testing positive**	21/33 (64%)	10/12 (83%)	0.29
**CFTR**	7/31 (23%)	7/11 (64%)	0.02
**PRSS1**	8/31 (26%)	1/12 (8%)	0.41
**SPINK1**	10/30 (33%)	2/11 (18%)	0.46
**CTRC**	0/21 (0%)	1/8 (13%)	0.28
**ARP at DM testing (yes)**	21 (54%)	9 (69%)	0.52
**Time ARP to DM testing (years)**	0.6 (0.2, 1.5)	1.0 (0.1, 1.6)	0.72
**CP at DM testing (yes)**	13 (33%)	6 (46%)	0.51
**Time CP to DM testing (years)**	0.2 (0.0, 0.9)	0.1 (0.0, 0.4)	0.66

Data presented as median (25^th^, 75^th^ percentile) and frequency (%).

*Considered abnormal if: fasting glucose was ≥ 100 mg/dL or 2 hour glucose was ≥ 140 mg/dL or HbA1c was ≥ 5.7%.

Glucose and HbA1c testing were not done regularly and most did not have their first screening until after their 2^nd^ AP attack for the ARP patients. First abnormal test on record or if no record of abnormal testing then first normal test. BMI (body mass Index), SAP (Severe Acute Pancreatitis), ARP (Acute Recurrent Pancreatitis), DM (Diabetes Mellitus), CP (Chronic Pancreatitis).

In an attempt to investigate the type of DM, we reviewed the islet auto antibodies results and found them to be negative in all patients with abnormal testing suggesting against a Type 1 DM subtype. Fasting insulin was normal (<20mIU/L) in all patients tested in the normal glucose group (54% had insulin testing (21/39), normal fasting insulin 21/21). In the abnormal glucose group, 46% (6/13) had insulin testing and 83% (5/6) of those tested had normal fasting insulin, a high fasting insulin in 1 patient suggesting possibly a Type 2 DM.

### Gene variant distribution in normal versus abnormal glucose testing groups

Genetic tests were obtained as per the treating provider, we had data available on which genes were tested, and variants found in those tested. The most commonly tested genes were: Cationic Trypsinogen gene *(PRSS1*), Cystic Fibrosis Transmembrane Regulator *(CFTR)* gene, Serine Protease Inhibitor, Kazal-Type 1 (*SPINK1)* gene, Chymotrypsin C (*CTRC*) gene.

**Fig 1:**
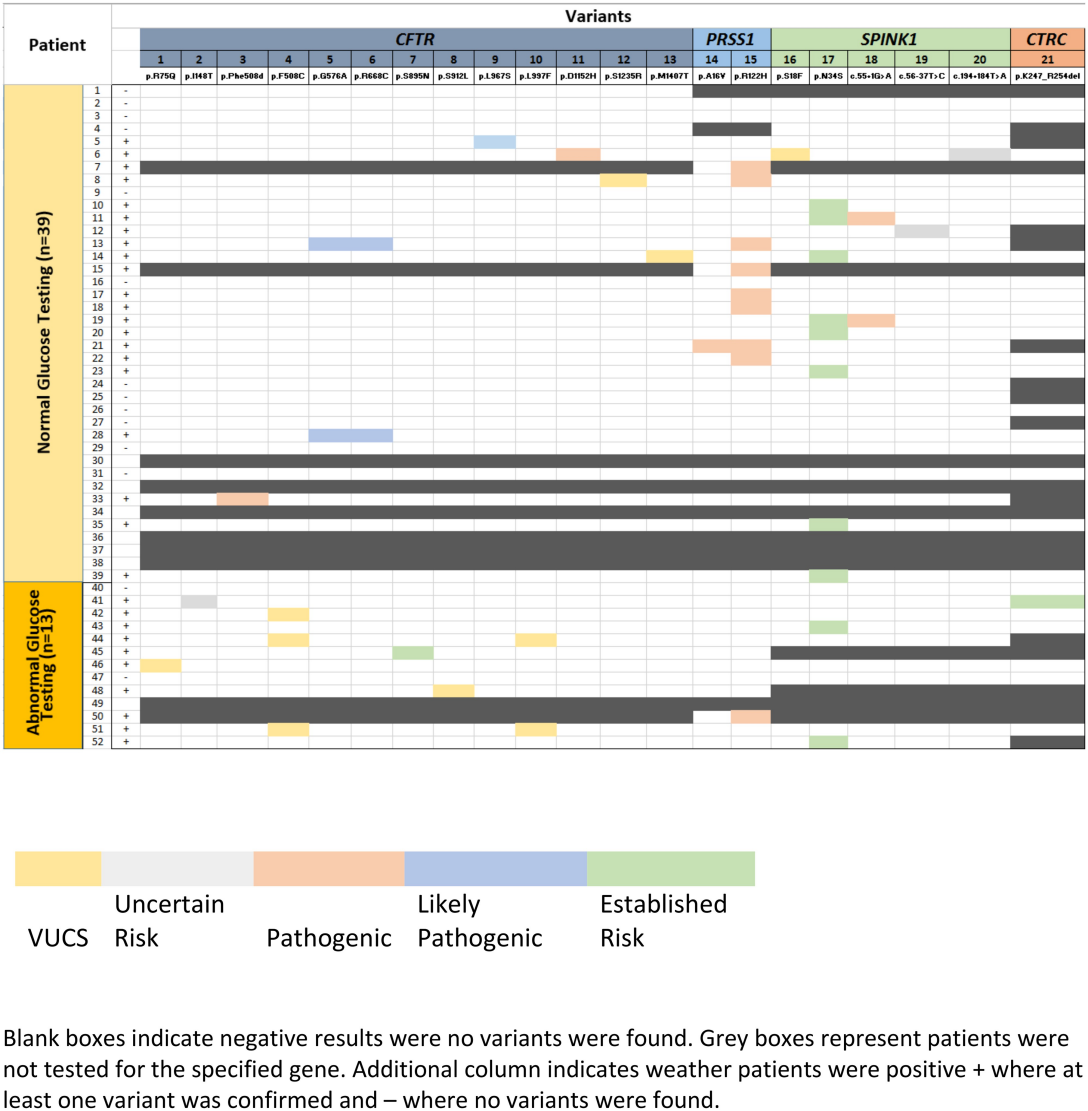
Heatmap of genetic variant distribution in the normal and the abnormal glucose testing groups. **Gene variants identified after a rigorous filtering process are shown as a heat map**. Specific *CFTR* variants are associated with abnormal glucose metabolism post-AP (p = 0.02), by Fisher Exact test. The individual patients represented as rows (n = 52). The unique variants that were confirmed in each patient are represented as columns (n = 21). Variants found in the individual patients as heterozygous are represented by the boxed highlights. The variants are classified into 5 categories each category is represented by a different color as follows.

Using a gene level analysis we showed that specific *CFTR* variants are associated with abnormal glucose metabolism post-AP (p = 0.02), while the other genes (PRSS1, SPINK1, and CTRC) were not significantly associated with abnormal glucose testing, Table 2. Specific variants distributions between the normal and the abnormal glucose testing groups are summarized in the Heatmap as shown in Fig 1. Table 3 lists the variants found in each group, the normal and the abnormal glucose testing group. In the total cohort (normal and abnormal glucose metabolism), a large number of variants (n = 13) of reportable classification were detected in the *CFTR* gene. Of 13 variants, 5 were classified as likely pathogenic or pathogenic and an equal number were in the VUCS category. *SPINK1* had the next largest number of variants, namely, 5, followed by *PRSS1* (n = 2, both pathogenic) and *CTRC* (n = 1, established risk). *SPINK1* only had one pathogenic and one VUCS variant. The rest of the changes were risk variants.

**Table 3:** List of genetic variants in normal and abnormal glucose testing.

Variants	Gene	Mutations	No. Pts Normal Glucose Metabolism (positive/tested)	No. Pts. Abnormal Glucose Metabolism (positive/tested)	Patients	Controls (gnomAD Database) Allele Count/ Allele Number (%)	Classification
1	CFTR	p.R75Q (het)	0/31	1/11	1/42	4306/276678 (1.56)	VUCS
2		p.I148T (het)	0/31	1/11	1/42	469/276270 (0.17)	Uncertain Risk
3		p.Phe508del (het)	1/31	0/11	1/42	1947/276982 (0.7)	Pathogenic
4		p.F508C (het)	0/31	3/11	3/42	250/273092 (0.09)	VUCS
5		p.G576A (het)	2/31	0/11	2/42	1375/276228 (0.5)	Likely Pathogenic
6		p.R668C (het)	2/31	0/11	2/42	1630/275868 (0.59)	Likely Pathogenic
7		p.S895N (het)	0/31	1/11	1/42	87/277018 (0.03)	Established Risk
8		p.S912L (het)	0/31	1/11	1/42	274/277106 (0.1)	VUCS
9		p.L967S (het)	1/31	0/11	1/42	192/276960 (0.07)	Likely Pathogenic
10		p.L997F (het)	0/31	2/11	2/42	611/276606 (0.22)	VUCS
11		p.D1152H (het)	1/31	0/11	1/42	104/276606 (0.04)	Pathogenic
12		p.S1235R (het)	1/31	0/11	1/42	1368/273990 (0.5)	VUCS
13		p.M1407T (het)	1/31	0/11	1/42	NR (0)	VUCS
14	PRSS1	p.A16V (het)	1/31	0/12	1/52	1680/256738 (0.65)	Pathogenic
15		p.R122H (het)	8/31	1/12	9/52	NR (0)	Pathogenic
16	SPINK1	p.S18F (het)	1/30	0/9	1/52	NR (0)	VUCS
17		p.N34S (het)	8/30	2/9	10/52	2498/275370 (0.91)	Established Risk
18		c.55+1G>A (het)	2/30	0/9	2/52	NR (0)	Pathogenic
19		c.56-37T>C	1/30	0/9	1/52	2279/275032 (0.83)	Uncertain Risk
20		c.194+184T>A (het)	1/30	0/9	1/52	366/30970 (1.18)	Uncertain Risk
21	CTRC	p.K247_R254del (het)	0/21	1/7	1/52	21/277012 (0.01)	Established Risk

gnomAD: The Genome Aggregation Database established by Broad Institute (http://gnomad.broadinstitute.org/). NR: Not Reported in the gnomAD. VUCS: variant of uncertain clinical significance. At the time of testing, the laboratory lacks conclusive evidence (neither positive nor negative) to determine the gene change is harmful which increases disease risk. Further re-evaluation of new evidence is expected.

## Discussion

Our study is innovative since it examines the effect of clinical variables and genetics on pre-DM as a risk for DM post AP, an approach that has not been taken before in pediatrics. This is the first study that investigated the timing of glucose abnormalities in children post pancreatitis. We have shown that older age, overweight/obesity BMI percentile and the presence of SAP were associated with an increased risk of pre-DM or DM post AP. Older age having a greater risk of pre-DM and DM abnormality may be related to the fact that these cases may have been associated with subtle attacks of pancreatitis in the past, a phenomenon that we are unable to elucidate or confirm from our data set. We analyzed SAP and overweight/obesity in all AP patients and it was not different in distribution from the patients that underwent glucose testing (unpublished data). Given that the testing was performed at varying intervals post AP, we are unable to discern if this is a transient or a sustained phenomenon, or the timing of onset. Patients were tested either by HbA1c, fasting blood glucose (FBG) or oral glucose tolerance testing (OGTT). While it remains unclear which test is optimal for pancreatogenic diabetes screening,[14] all patients who had abnormal OGTT also had either an abnormal HbA1c or FBG. The findings of negative islet autoantibodies and normal insulin values in the majority of patients, suggests that T3cDM as a possible cause of these abnormalities. While we cannot yet conclude which diabetes subtype is occurring in our pancreatitis patients from this retrospective study, our goal from this analysis is to first identify the group that is at risk for glucose abnormalities, to allow further research testing to study if this is Type 1, Type 2 or Type 3c DM in the future[24].

From a gene level analysis, we showed that specific *CFTR* variants were associated with abnormal glucose metabolism post-AP. A limitation of this data is that only a limited number of genes were tested and not systematically in all patients. Future expanded genetic testing will emphasize the role of personalized medicine in predicting the phenotype.

DM does not present with clear symptoms, so it can be overlooked clinically resulting in adverse implications if untreated [25–27]. DM occurs post pediatric CP [13] and from our studies we showed that it can occur following AP as early as the first attack in certain patient subsets. DM in pancreatitis patients has been linked to increased risk of pancreatic adenocarcinoma highlighting further the importance of recognizing it early on by adequate screening methods [3, 15, 16, 28]. We lack pediatric studies that comprehensively analyze factors leading to abnormal glucose tests after AP, resulting in a lack of appropriate screening for post-AP DM. To design therapies that prevent progression to DM, we need to elucidate the observable and targetable underlying clinical and genetic traits using a longitudinal approach beginning at each patient’s clinical presentation with AP.

While our study is the first to highlight the prevalence of abnormal glucose testing post AP, it has limitations. The retrospective nature and the non-systematic screening for DM, does not allow for a precise understanding of the initiating factors. From our current study, we may not be able to conclude whether DM is a transient or sustained effect in the first 1–2 years, and it would be important to design prospective studies that allow us to examine this trajectory.

## In conclusion

In our patient population, abnormal glucose testing was noted as early as after the first attack, and a higher proportion of glucose abnormalities were identified after the second AP attack even in the absence of CP. We found older age, overweight/obesity and having SAP during the prior AP episode to be associated with abnormal glucose testing. In order to corroborate our findings, we propose studies that allow for systematic screening for abnormal glucose after the first AP episode in order to better establish the timing of onset of diabetes and its progression.
